# Pediatric lichen sclerosus: a self-limited disease? A long-term follow-up case series^؆^

**DOI:** 10.1016/j.abd.2026.501394

**Published:** 2026-06-16

**Authors:** Carlos M. Nogueira, Filomena Azevedo, Carmen Lisboa

**Affiliations:** a Dermatovenereology Department, Unidade Local de Saúde de Braga, Braga, Portugal; b Dermatovenereology Department, Unidade Local de Saúde de São João, Porto, Portugal; c RISE-Health, Pathology Department, Faculty of Medicine, Universidade do Porto, Porto, Portugal

Dear Editor,

Lichen Sclerosus (LS) is a chronic inflammatory dermatosis of unknown etiology with a predilection for the anogenital region. In females, the disease incidence follows a bimodal distribution, with a more pronounced peak after menopause and a second in prepubertal girls.^1^ Female pediatric patients most commonly present with vulvar pruritus, discomfort, fissures, and sometimes constipation due to perianal involvement.^1^, ^2^ Although less commonly reported, the disease also occurs in prepubertal boys, who may present with hypopigmented plaques on the prepuce or glans, or, most commonly, as acquired scarring phimosis.^2^, ^3^ Diagnosis is frequently delayed or missed in both cases.^4^ The disease is usually responsive to potent topical steroids, with some clinicians advocating the use of topical calcineurin inhibitors.^2^ There are currently no standardized treatment protocols for the pediatric population, and the long-term evolution of lichen sclerosus diagnosed in childhood remains poorly understood, with a striking lack of longitudinal studies exploring its natural history and persistence beyond puberty.

We conducted a retrospective case series at a tertiary dermatology department in Portugal, aiming to describe the clinical course of pediatric-onset genital LS into adulthood. Inclusion criteria were diagnosis before age 18 and prior to 2010, and availability of sufficient clinical data and/or successful contact for follow-up. Cases were identified through departmental archives, and medical records were reviewed for clinical data. Additional information was obtained from other centers and/or primary care facilities. All participants were contacted by phone to assess their current status. A structured interview collected data on symptom recurrence, anatomical changes, additional interventions/consultations, need for treatment, and psychosocial impact. Follow-up consultations were offered to all. Verbal informed consent was obtained from all participants.

Five participants were included: three females and two males. Mean age at LS diagnosis was 11-years (range 10–13y). Symptoms had been present one to three years prior to diagnosis. Four cases were confirmed histologically. Table 1 summarizes key clinical data. Initial symptoms included mainly hypopigmented, atrophic, or fissured plaques, with pruritus or pain. A 10-year-old boy presented with scarring phimosis, evolving for over three years. All patients were initially treated with high-potency topical corticosteroids (betamethasone dipropionate 0.5 mg/g), and maintenance therapy included intermittent topical corticosteroids or calcineurin inhibitors (e.g., tacrolimus 0.03% ointment and pimecrolimus 1% cream). There were no reported treatment-related side effects. All the cases achieved clinical remission during childhood and were discharged or lost to follow-up before puberty.

**Table 1:** Clinical characteristics, treatment, and long-term outcomes of patients with pediatric-onset lichen sclerosus.

	Age at diagnosis (years)	Sex	Time to diagnosis (years)	Initial Symptoms	Histopathological examination	Remission during childhood	Relapse during/after puberty	Complications
Case 1	10	Female	2	Pruritus, atrophic whitish plaques and purpura on the vulva and perianal region	Yes	Yes	**No**	None
Case 2	12	Female	1	Pruritus, atrophic whitish plaques on the vulva	Yes	Yes	**Yes**	None
Case 3	10	Female	2	Atrophic whitish plaques on the vulva	Yes	Yes	**Yes**	None
Case 4	13	Male	2	Atrophic whitish plaques on the glans and prepuce	No	Yes	**No**	None
Case 5	10	Male	3	Scarring phimosis	Yes	Yes	**Yes**	Recurrent phimosis, Meatal Stenosis, Urethral Stricture, Depression, Suicide Attempt, Extragenital lesions

After a mean follow-up period of 16.2-years (range: 13–21y), only two participants reported complete, sustained remission and three experienced disease recurrence during/after puberty. Two of these were females, who resumed specialized follow-up elsewhere, as well as topical corticosteroid therapy, achieving symptom control. Neither of them reported anatomical changes. The remaining case had recurrent balanitis beginning in adolescence (Fig. 1). Over time, he developed urethral narrowing requiring multiple urological interventions, including urethrotomy, urethroplasty, several urethral dilatations and correction of an acquired fibrotic preputial ring. These surgeries resulted in postoperative urinary incontinence. This patient required psychiatric support due to associated depression and a suicide attempt.

**Fig. 1:**
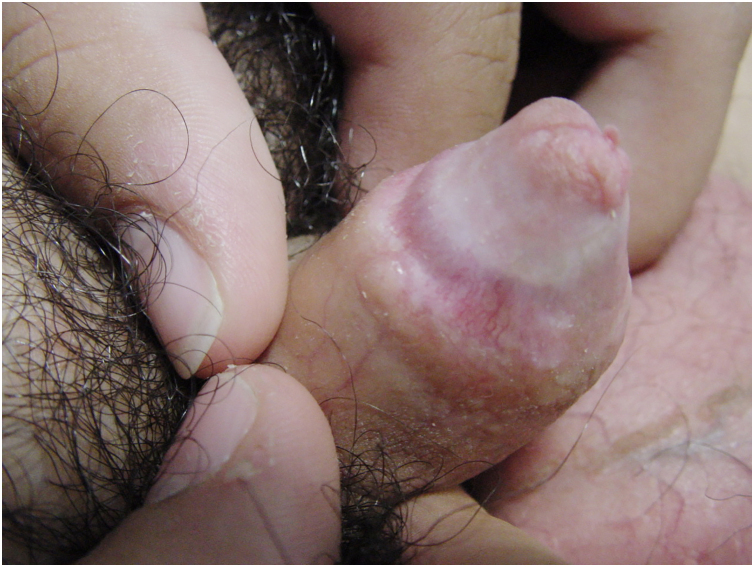
Sclerosis and atrophy of the entire glans, with telangiectasias, stenosis of the urethral meatus, and complete obliteration of the balanopreputial sulcus.

Our study found that three out of five pediatric LS cases followed until adulthood had recurrent disease beyond puberty. In adults, it is well established that LS is a relapsing and remitting dermatosis with a chronic course. However, traditionally, LS in children has been considered a transient condition, expected to resolve spontaneously with puberty. This belief has been increasingly challenged by a growing body of evidence pointing otherwise.^5^, ^6^, ^7^, ^8^, ^9^

This case series highlights the chronic potential of childhood LS. Although initial remission was achieved in all cases, recurrence during adolescence or early adulthood occurred in the majority. Despite our small sample size, these findings align with several long-term studies. Smith et al. demonstrated persistent symptoms and need for ongoing treatment in 75% of 18 adolescent girls diagnosed with LS before puberty;^6^ another case series with 15 female patients by Patrizi et al. also found a high relapse rate, with 60% of girls relapsing during follow-up and 20% with permanent anatomical sequelae.^7^ Focseneanu et al. also reported recurrence in almost half of their cohort of premenarchal girls (44%), particularly if active disease was present at menarche.^8^ However, this study didn’t require follow-up until/after puberty, and only three of the documented recurrences occurred in post-menarchal patients. Additionally, an important limitation of our study was the use of telephone interviews for follow-up assessment, which may have failed to detect subtle or minimally symptomatic disease and therefore potentially underestimated post-pubertal recurrence rates.

Our recurrence rate of 3/5 and anatomical complication rate of 1/5 are consistent with this scarce literature. Additionally, to our knowledge, this is the first case series of pediatric LS with follow-up until adulthood that includes male patients. Notably, the male patient with progression to urethral stricture and other complications had a particularly delayed diagnosis, reflecting the potential severity of untreated or unmonitored LS in boys, a group often underrepresented in studies. Delay in diagnosis, ranging from 1 to over 3 years, was also common in our series, consistent with published findings.^9^ Misdiagnosis or minimization of symptoms may contribute to this delay, and early referral to dermatology or pediatric gynecology/urology is key. The shared presentation of hypopigmented genital lesions makes vitiligo a key differential diagnosis in this age group; however, vitiligo is usually asymptomatic and lacks textural change, underscoring the importance of careful clinical evaluation when histopathology is not feasible.

While LS is often responsive to topical corticosteroids, the optimal treatment regimen remains undefined. Our results support the safety and effectiveness of potent corticosteroids for both induction and maintenance therapy, as well as topical calcineurin inhibitors, without reported adverse effects. However, the use of topical calcineurin inhibitors remains controversial.^10^ Importantly, expecting a self-limited disease can lead to undertreated or inadequately followed LS, contributing to irreversible complications, including labial fusion, clitoral burying, and, in both sexes, urethral stenosis.

The psychosocial burden of chronic genital disease is difficult to quantify but was also evident in our cohort, with severe depression and attempted suicide in one case. This underscores the importance of a multidisciplinary approach, including psychological support.

Currently, there is no consensus on specific follow-up orientations for children, and it remains unclear when to discharge or how to monitor patients during and after puberty. Clinicians should be cautious about discharging patients prematurely. Based on our findings, we advocate for continued follow-up into adolescence and young adulthood, especially in patients with active disease or poor treatment adherence.

## Authors' contributions

Carlos M. Nogueira: Conceptualization, data collection, patient interviews, analysis and interpretation of data, and drafting of the manuscript.

Filomena Azevedo: Supervision and critical review of the manuscript.

Carmen Lisboa: Conceptualization, supervision, critical revision of the manuscript, and patient interviews.

All authors contributed to the final version of the manuscript, approved it for submission, and agree to be accountable for all aspects of the work.

## Financial support

None declared.

## Research data availability

The entire dataset supporting the results of this study was published in this article.

## Conflicts of interest

None declared.
